# ΔFW-NPS6-Dependent Transcriptome Profiling Reveals Putative Pathogenicity Genes in *Fusarium oxysporum*

**DOI:** 10.3390/ijms27020830

**Published:** 2026-01-14

**Authors:** Xuhong Ye, Li Zhang, Jianjie Zhang, Haozhe Lu, Jiaqi Li, Hongtao Zou

**Affiliations:** 1 College of Land and Environment, Shenyang Agricultural University, Dongling Road 120, Shenyang 110866, China; 2 National Engineering Research Center for Efficient Utilization of Soil and Fertilizer Resources, Shenyang 110866, China; 3 Northeast Key Laboratory of Conservation and Improvement of Cultivated Land (Shenyang), Ministry of Agriculture and Rural Affairs, Shenyang 110866, China

**Keywords:** nonribosomal peptide synthetase 6, RNA-Seq, pathogenic gene, QPCR, fusarium oxysporum

## Abstract

*Fusarium oxysporum* f. sp. *niveum* is an increasingly threatening fungal pathogen that systemically colonizes watermelon plants and severely compromises their productivity by causing destructive vascular wilt disease. While its nonribosomal peptide synthetase NPS6 has been identified as a key virulence factor, the regulatory mechanisms through which it controls downstream gene networks to cause disease remain unclear. To elucidate this regulatory pathway, we constructed a ΔFW-NPS6 knockout mutant and conducted a comparative genome-wide analysis using RNA sequencing, with the wild-type strain as a control. The results revealed 66 NPS6-dependent differentially expressed genes, which were primarily associated with secondary-metabolite biosynthesis (e.g., genes encoding nonribosomal peptide synthetases like NPS2) and pathogen–host interactions (e.g., components of the MAPK signaling pathway), and were enriched in key pathogenic pathways. This finding reveals the virulence regulatory network mediated by NPS6, providing a direct theoretical foundation and crucial molecular targets for developing novel control strategies, such as targeted fungicides or genetic interventions, against Fusarium wilt in watermelon by highlighting NPS6 itself as a potential fungicide target and its downstream pathways (e.g., siderophore biosynthesis) as points for intervention.

## 1. Introduction

Fusarium wilt, a prevalent and devastating plant vascular disease, results from fungal infections that systematically colonize the host plant’s vascular tissues [1]. The primary causative agent of this disease is *Fusarium oxysporum*, a species characterized by significant pathogenic specialization. It is further classified into multiple formae speciales (abbreviated as f. sp.), each typically comprising one or several physiological races defined by their capacity to infect specific host plants [2]. Among these, *Fusarium oxysporum* f. sp. *niveum* (FON) is particularly notable for its specific pathogenicity toward watermelon (*Citrullus lanatus*) [3]. This pathogen poses a severe threat to watermelon crops across all developmental stages, leading to substantial yield losses and has remained a persistent challenge for fruit growers worldwide [4].

Currently, control strategies for Fusarium wilt in watermelon are primarily developed based on the biological characteristics of different pathogenic races (Races 0, 1, and 2), including practices such as grafting susceptible scions onto resistant rootstocks and employing biological control agents [5,6]. hypothesized, due to the complexity and variability of the mechanisms underlying the pathogen’s ability to cause disease and, more importantly, the limited understanding of its pathogenicity at the molecular level, current control strategies still fail to address the fundamental cause of the disease, making sustainable control elusive, namely, the lack of knowledge about master regulators and their core virulence networks such as those governed by NPS6, making sustainable control elusive. Therefore, elucidating the molecular mechanisms governing fungal virulence, particularly the role of key pathogenicity determinants such as nonribosomal peptide synthetase (NPS), is essential for developing effective and durable control solutions [7]. These constraints underscore the imperative to conduct in-depth molecular-level investigations into the virulence factors of FON to facilitate the development of novel, targeted, and sustainable management strategies.

At the molecular level, numerous pathogenicity-associated factors have been identified in *Fusarium oxysporum*, revealing a suite of sophisticated cellular responses during infection. These include complex signal transduction pathways [8,9], the secretion of cell wall–degrading enzymes, and diverse mechanisms that counteract or evade plant immune defenses [10]. However, existing knowledge remains largely fragmented, offering a catalog of components without a clear understanding of the regulatory circuitry that coordinates them. The major bottleneck is no longer the shortage of candidate genes but the absence of a systems-level view of how these elements are hierarchically organized and functionally integrated. As a result, the rational design of next-generation control strategies, such as targeted antifungals or host-induced gene silencing, is significantly hindered by the inability to identify master regulators and their core downstream networks, which represent the most effective and durable points of intervention.

Nonribosomal peptide synthetases (NPSs), recognized as key virulence factors in a wide range of fungal pathogens [11,12], can undergo post-assembly modifications such as glycosylation, methylation, and acylation, thereby expanding their functional diversity [13]. In plant-pathogenic fungi, NPS-derived metabolites have been demonstrated to serve multiple biological functions. These include acting as signaling molecules that regulate developmental processes, functioning as phytotoxins that directly damage plant tissues, assisting in the degradation of host components, contributing to resistance against host-derived oxidative stress, serving as siderophores for iron acquisition under iron-limited conditions, and acting as virulence effectors that modulate host immunity to promote infection [14,15,16]. Consequently, NPSs are widely regarded as central elements in the pathogenesis strategies of numerous plant-pathogenic fungi. Among the NPSs, NPS6 has attracted particular interest as a key virulence factor because of its well-established roles in siderophore biosynthesis, oxidative stress tolerance, and the regulation of fungal virulence [17,18,19]. Functionally, NPS6 is responsible for the synthesis of fusarinine-type siderophores, which are secreted to chelate extracellular iron, a critical step for successful host colonization and infection [20,21,22]. Studies in other fungal pathogens, such as *Alternaria alternata* and several ascomycetes, have demonstrated that NPS6 participates in siderophore-mediated iron acquisition [11,23]. Notably, genetic disruption of NPS6 in these fungi results in significant reductions in virulence and increased sensitivity to reactive oxygen species [11,24], highlighting its conserved role in mitigating host-induced oxidative stress. While our recent study confirmed that NPS6 is essential for full virulence in FON [1], the specific regulatory network it governs remains uncharacterized. However, despite these advances in related fungal systems, the specific functions of NPS6 and its associated virulence-regulatory networks in *Fusarium oxysporum* remain largely unknown. Based on its conserved role in siderophore biosynthesis and oxidative stress tolerance, we hypothesized that NPS6 is a master virulence regulator in FON. We specifically predicted that the deletion of NPS6 would not only impair iron homeostasis and redox balance but also lead to a broad transcriptional reprogramming. This reprogramming was anticipated to affect key pathogenic processes, including the biosynthesis of secondary metabolites and the modulation of signaling pathways, thereby compromising the ability of the fungus to colonize and infect watermelon plants.

To test this hypothesis and fill this knowledge gap, we employ genetic manipulation and pathogenicity assays to determine whether these candidate genes mediate the virulence function of NPS6, thereby clarifying their roles in the pathogenic mechanisms of this economically important fungus. To achieve this goal, we compare the genomic and transcriptomic profiles of wild-type and NPS6-deficient strains through comprehensive RNA sequencing and bioinformatics analyses to identify potential virulence factors functionally associated with NPS6. Through these analyses, we seek to delineate the NPS6-centered pathogenic regulatory network and provide insights and experimental evidence for understanding the systemic pathogenic mechanisms of *Fusarium oxysporum* f. sp. *niveum*.

## 2. Results

### 2.1. Distribution of Mapped Reads

To comprehensively characterize the transcriptional profiles of the wild-type (WT) *Fusarium oxysporum* f. sp. *niveum* and the ΔFW-NPS6 mutant strain, high-throughput cDNA libraries were constructed and subjected to RNA sequencing. After quality control and filtering, a total of 4,782,664 and 5,047,895 raw sequence reads were generated for the WT and ΔNPS6 libraries, respectively. From these, 2,378,325 and 2,540,752 unique reads were successfully mapped to the reference genome for the WT and mutant strains, indicating robust sequencing depth for subsequent differential expression analysis. A substantial proportion of these sequence reads were successfully annotated, accounting for 59.78% (1,421,151 reads) of the WT and 45.55% (1,246,324 reads) of the ΔFW-NPS6 transcriptomes. Analysis of gene expression levels, quantified using the RPKM (Reads Per Kilobase of transcript per Million mapped reads) metric, revealed that the majority of genes exhibited moderate expression levels. Specifically, 470 genes in the WT and 580 in the mutant were expressed within an RPKM range of 1.0 to 50. In contrast, only a small subset of genes demonstrated very high expression levels, with 7 genes in the WT and 13 in ΔFW-NPS6 having RPKM values exceeding 500.

### 2.2. Gene Ontology (GO) Classification

**Figure 1:**
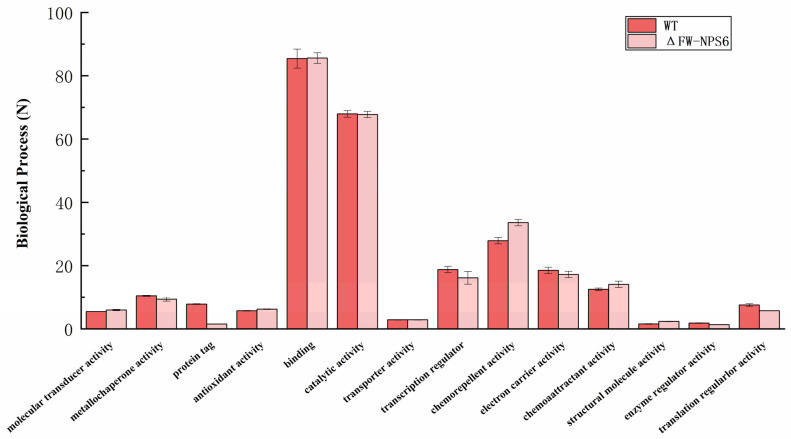
Biological process of Gene Ontology classification of annotated genes in wild-type (WT) and ΔFW-NPS6 mutant strains. The two strains showed no significant differences in their classification hierarchies (*p* > 0.05).

**Figure 2:**
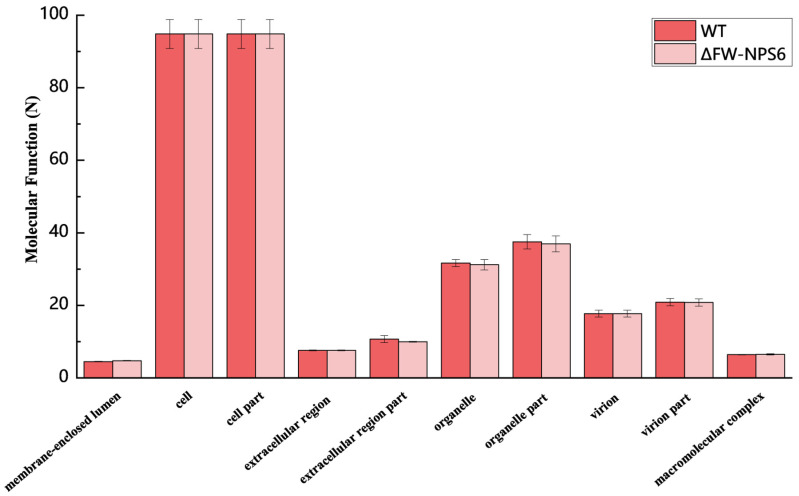
Molecular function of Gene Ontology classification of annotated genes in wild-type (WT) and ΔFW-NPS6 mutant strains. The two strains showed no significant differences in their GO classification hierarchies (*p* > 0.05).

**Figure 3:**
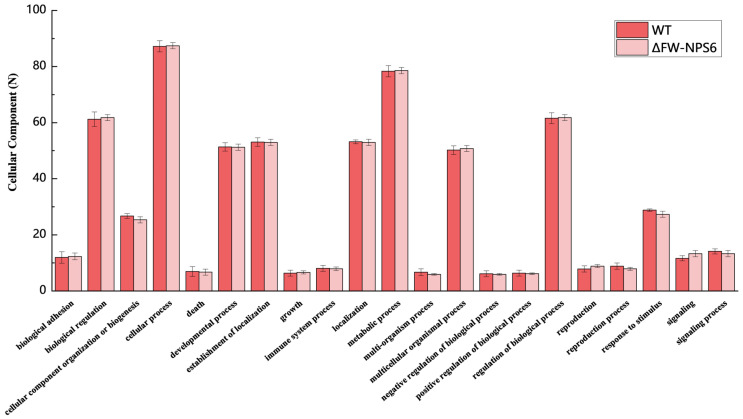
Cellular component of Gene Ontology classification of annotated genes in wild-type (WT) and ΔFW-NPS6 mutant strains. The two strains showed no significant differences in their classification hierarchies (*p* > 0.05).

To gain functional insights into the transcriptomes of the wild-type (WT) *Fusarium oxysporum* f. sp. *niveum* and the ΔFW-NPS6 mutant, we performed a systematic functional classification of the annotated genes. Gene Ontology (GO) analysis was used to categorize the predicted genes into three major categories: biological process, molecular function, and cellular component. The overall distribution of genes within the principal level-2 GO terms for each of these categories is visually summarized in Figure 1 (biological process), Figure 2 (molecular function), and Figure 3 (cellular component). The absence of major shifts in the global functional profiles (Figure 1, Figure 2 and Figure 3) indicates that the core cellular machinery remains largely intact in the ΔNPS6 mutant under the conditions tested. This conservation of baseline functions underscores the specificity of the transcriptional reprogramming revealed by the subsequent differential expression analysis, highlighting pathways directly influenced by NPS6.

From the total set of expressed genes, 1634 from the WT and 1672 from the ΔFW-NPS6 mutant were subjected to GO analysis. Among these, a high percentage—1297 (78.41%) genes for the WT and 1311 (79.38%) for the mutant—were successfully assigned GO terms. The distribution of these annotated genes across the three principal GO categories was as follows: for the WT, 743 genes were linked to Biological Process, 1130 to Molecular Function, and 550 to Cellular Component. Correspondingly, for the ΔFW-NPS6 mutant, 733, 1021, and 721 genes were annotated in these same categories, respectively. Notably, the overall functional classification profile was highly conserved between the two strains, with no significant differences observed at this level of analysis. A more granular examination at level 2 further subdivided the genes into 53 distinct functional groups for both the WT and mutant. Within the Biological Process domain, the subcategories cellular process and metabolic process constituted the predominant functional classes. For the Cellular Component category, the terms cell part and cell were the most heavily represented. In the Molecular Function category, binding was the most abundant activity, followed by catalytic activity.

Complementing the GO analysis, pathway assignment and functional classification were also carried out using the Kyoto Encyclopedia of Genes and Genomes (KEGG) database. This analysis revealed that 536 genes from the WT and 542 genes from the ΔFW-NPS6 mutant were assigned one or more KEGG annotations and could be mapped to 201 distinct KEGG pathways (Table 1). The proportional distribution of these annotated genes into major KEGG categories was remarkably similar between the two strains. In the WT, the breakdown was 25.40% for Cellular Processes, 19.88% for Environmental Information Processing, 43.78% for Metabolism, and 7.34% for Genetic Information Processing. The ΔFW-NPS6 mutant exhibited a nearly identical profile, with 25.08%, 20.09%, 43.37%, and 7.56% of its genes classified into these same categories, respectively. A closer inspection of the Cellular Processes category indicated that the majority of genes in both strains were involved in functions pertaining to the immune system, cell communication, and the endocrine system. Within the overarching Metabolism category, pathways for carbohydrate metabolism, amino acid metabolism, and lipid metabolism were most prominently represented.

**Figure 4:**
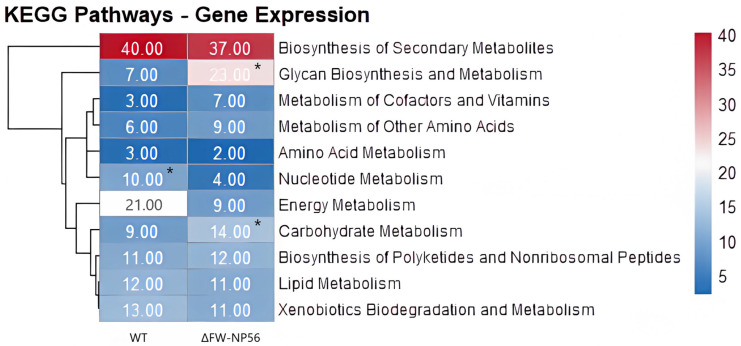
Heatmap Analysis of KEGG Metabolic Pathway Gene Expression in WT and ΔFW-NP56 Strains. “*” indicates a statistically significant difference (*p* < 0.05) in gene count between WT and ΔFW-NPS6 strains.

To evaluate the impact of FW-NPS6 deletion at the metabolic pathway level, we conducted a comparative analysis of genes assigned to KEGG metabolic pathways in the wild-type (WT) and ΔFW-NPS6 mutant strains, with the results visualized in a heatmap (Figure 4). Statistical analysis confirmed that the reductions in gene counts associated with “Energy Metabolism” (WT: 21 vs. ΔFW-NPS6: 9) and “Nucleotide Metabolism” (WT: 10 vs. ΔFW-NPS6: 4) were statistically significant (*p* < 0.05). In contrast, “Carbohydrate Metabolism” showed a significant increase in gene number (WT: 9 vs. ΔFW-NPS6: 14). No significant differences were observed in pathways including “Biosynthesis of Secondary Metabolites” (WT: 40 vs. ΔFW-NPS6: 37), “Biosynthesis of Polyketides and Nonribosomal Peptides” (WT: 11 vs. ΔFW-NPS6: 12), and “Lipid Metabolism” (WT: 13 vs. ΔFW-NPS6: 11). These results demonstrate that FW-NPS6 deletion specifically impairs fundamental energy and nucleotide metabolism while preserving the biosynthetic capacity for secondary metabolites, indicating its crucial role in regulating core energetic processes and cellular building block supply.

### 2.3. Identification of the Differentially Expressed Genes

To systematically identify genes whose expression was influenced by the deletion of the NPS6 gene, we conducted a comparative transcriptome analysis between the wild-type (WT) *Fusarium oxysporum* f. sp. *niveum* and the ΔFW-NPS6 mutant. A stringent screening criterion, requiring a *p*-value < 0.01 and an absolute fold-change > 2, was applied to ensure the high confidence of the results. This analysis revealed a total of 578 differentially expressed genes (DEGs). Among these, 279 genes were significantly upregulated in the WT strain relative to the mutant, suggesting their expression may be suppressed by or dependent on a functional NPS6. Conversely, 299 genes were downregulated in the WT, indicating their potential induction in the ΔFW-NPS6 genetic background. Following this identification, a crucial step of functional annotation was performed. We successfully annotated 162 of the upregulated genes and 128 of the downregulated genes, providing them with definitive gene names and functional descriptions, which form a critical foundation for subsequent mechanistic interpretations. Notably, the significant downregulation of genes involved in energy and nucleotide metabolism in the ΔNPS6 mutant suggests that NPS6 may play a previously underappreciated role in regulating core metabolic processes, which could be crucial for supplying the energy and building blocks required for host invasion.

**Figure 5:**
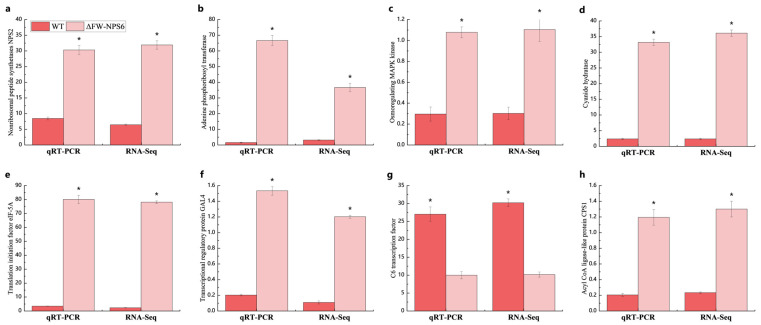
qRT-PCR validation of 8 genes that were differentially expressed between WT and ΔFW-NPS6. (**a**) Nonribosomal peptide synthetases NPS2; (**b**) Adenine phosphoribosyl transferase; (**c**) Osmoregulating MAPK; (**d**) Cyanide hydratase; (**e**) Translation initiation factor eIF-5A; (**f**) Transcriptional regulatory protein GAL4; (**g**) C6 transcription factor; (**h**) Acyl CoA ligase-like protein CPS1. An asterisk (*) indicates a significant difference among treatments at the *p* < 0.05 level.

To independently verify the reliability and accuracy of the RNA-Seq data, a subset of ten DEGs was randomly selected for technical validation using quantitative real-time PCR (qRT-PCR). The expression trends and magnitudes of change for these selected genes, as determined by qRT-PCR, showed a strong and statistically significant correlation with the expression profiles generated from the RNA-Seq analysis. This high degree of consistency between the two methodologies, as graphically depicted in Figure 5, confirms the technical robustness of our transcriptome sequencing and the credibility of the identified DEGs for further biological investigation.

### 2.4. Identification of High-Confidence NPS6-Dependent Genes

These 66 genes constitute a high-confidence subset of the 578 DEGs, identified by applying an additional filter for expression abundance (RPKM > average). To systematically uncover novel genes functionally linked to the nps6-mediated virulence pathway in *Fusarium oxysporum* f. sp. *niveum*, we performed a targeted screening of our transcriptome data based on stringent expression criteria. The screening strategy was designed to identify genes not only exhibiting significant differential expression but also maintaining substantial expression levels, thereby increasing the likelihood of their biological relevance. Following the established methodology of Zheng et al. [25], we defined WT-enriched genes as those demonstrating a fold-change greater than 2 and possessing an RPKM value higher than the average RPKM of all genes in the WT background. Conversely, genes with a fold-change of less than −2 and an RPKM value exceeding the genetic background average were classified as ΔFW-NPS6-enriched. This dual-filter approach ensured the selection of high-confidence candidate genes. The resulting sets of enriched genes were subsequently subjected to in-depth functional prediction and pathway analysis using the KEGG Orthology (KO) database.

Application of this screening pipeline yielded 41 robustly WT-enriched genes and 54 ΔFW-NPS6-enriched genes. Within the WT-enriched gene set, 30 genes were successfully annotated with KO terms. Functional classification of these 30 genes revealed that the vast majority (27 genes) encoded various enzymes, while 3 genes were predicted to function as transcription factors, as detailed in Table 2. Parallel analysis of the 54 ΔFW-NPS6-enriched genes identified 36 with KO annotations. This group comprised 32 genes encoding enzymes and 4 genes for transcription factors. Notably, this set also included 4 genes belonging to the nonribosomal peptide synthetase (NRPS) gene family, highlighting a potential compensatory mechanism or an alternative secondary metabolite pathway activated in the absence of a functional NPS6. By integrating the candidate genes from both genotypes, this study successfully predicted a total of 66 putative pathogenic genes whose expression is strongly associated with the regulatory network of the NPS6 gene, providing a valuable resource for future functional characterization (Table 2). Among these, genes associated with secondary metabolite biosynthesis (particularly nonribosomal peptide synthetases like NPS2) and the MAPK signaling pathway emerge as particularly promising candidates for further experimental validation, given their well-established direct links to fungal pathogenesis and virulence regulation.

## 3. Discussion

*Fusarium oxysporum* f. sp. *niveum* (FON) is a devastating soil-borne facultative pathogen with a global distribution in major watermelon cultivation regions [5]. It incites a complex of destructive diseases, including vascular wilt, foot rot, and root and bulb rot, which collectively lead to substantial and often catastrophic reductions in crop yield and fruit quality, posing a persistent threat to global watermelon production. Given the well-established and diverse roles of nonribosomal peptide synthetases (NPSs) as critical virulence determinants-ranging from siderophore production to toxin biosynthesis in numerous plant-pathogenic fungi [11,12,26], they have been strongly proposed as key candidates mediating essential steps in plant pathogenesis. This study, therefore, sought to elucidate the specific molecular functions and regulatory network of a particular NPS gene, NPS6, in the pathogenesis of FON, aiming to uncover novel mechanisms that govern the pathogen‘s ability to cause disease. Unlike specific effector proteins such as FonSIX6 or transcription factors like FonPUF1 that execute discrete pathogenic functions, the NPS6-dependent regulon identified here may represent a broader regulatory layer that orchestrates fundamental metabolic and stress-responsive pathways necessary for successful infection. 

To systematically address this objective, we constructed and sequenced comprehensive cDNA libraries from both the wild-type (WT) strain and the isogenic ΔFW-NPS6 mutant. Subsequent high-quality functional annotation, comparative transcriptomics, and rigorous gene set enrichment analysis were employed to identify genes whose expression is associated with NPS6. Applying a stringent statistical threshold (*p*-value < 0.01, following the methodology of Wang et al., 2009 [25]) for defining DEGs, we identified 30 genes significantly enriched in the WT strain and 36 genes enriched in the ΔFW-NPS6 mutant. Bioinformatics analysis revealed that these candidate genes are implicated in a wide array of fundamental biological processes crucial for the fungal life cycle and host interaction. These processes encompass amino acid transport and metabolism [27,28], regulation of gene expression, secondary metabolite biosynthesis, transport and catabolism, carbohydrate transport and metabolism, cell cycle control, stress response, the MAPK signaling pathway [8,29], fatty acid and lipid metabolism, glycan biosynthesis and metabolism, inorganic ion transport and metabolism, and energy production and conversion (Table 2). The broad involvement across these diverse and critical functional categories strongly suggests their collective importance in the pathogenicity of FON, potentially under the direct or indirect regulatory influence of the NPS6 gene [30,31,32].

Amino acid transport and metabolism represent a key functional category identified in our study, with 6 WT-enriched and 7 ΔFW-NPS6-enriched genes implicated. This process is fundamentally important for fungal virulence, as it participates in critical developmental stages such as spore formation, mycelial and conidial morphogenesis, and the biosynthesis of mycotoxins like deoxynivalenol (DON) in various *Fusarium* species [5,25,33]. For instance, vacuolar amino acid transporters, exemplified by *FoAvt3p* in *F. oxysporum*, play a vital role in spore formation [25]. Similarly, enzymes in the branched-chain amino acid pathway, such as *FgIlv5* (acetohydroxy acid reductoisomerase) and *FgIlv1* (threonine dehydratase) in *Fusarium graminearum*, are required for isoleucine and valine biosynthesis and are indispensable for normal morphogenesis, DON production, and full pathogenic capability [5]. The identification of amino acid metabolism genes in our dataset suggests that NPS6-mediated virulence may involve the modulation of nitrogen metabolism and associated developmental pathways in FON, a link that warrants further functional characterization.

Iron homeostasis, mediated by siderophores, is another crucial virulence mechanism. Iron-chelating siderophores produced by fungal NPSs are integral to reproductive development, pathogenic processes, iron acquisition, and resistance to host-induced oxidative stress [30]. In *F. graminearum*, distinct roles for different NPSs have been elucidated: deletion of *NPS2* (intracellular siderophore biosynthesis) abolishes sexual sporulation, while deletion of NPS6 (extracellular siderophore biosynthesis) confers hypersensitivity to iron starvation and oxidative stress, leading to significantly reduced virulence, with evidence suggesting partial functional overlap between the two [34]. In our study, we observed a notable upregulation of *NPS2* in the ΔFW-NPS6 mutant strain. Given that both NPS2 and NPS6 are involved in siderophore metabolism but are responsible for distinct intracellular and extracellular pools, respectively [30], the upregulation of *NPS2* could represent an adaptive transcriptional response. This observation suggests a potential functional interaction or co-regulation within the NPS family that merits further investigation to determine if it partially offsets the metabolic deficiency caused by *NPS6* deletion. Conversely, other NPS genes, including *NPS4*, *NPS7*, *NPS9*, *NPS10*, and *NPS11*, were downregulated in the ΔFW-NPS6 strain. This suggests that their expression might be positively regulated, directly or indirectly, by a functional NPS6 in the WT background. While the product of *NPS4* has been hypothesized to function as a structural component of the cell wall or a regulator of surface hydrophobicity [10], the biological functions of *NPS7*, 9, 10, and 11 remain largely unknown [35], presenting intriguing targets for future research into the NPS6 regulon.

Signal transduction pathways, particularly the evolutionarily conserved mitogen-activated protein kinase (MAPK) cascades, serve as key regulators of pathogenesis in *Fusarium*. These pathways control a range of biological processes, including mating, conidiation, heterokaryon formation, host root penetration, and invasive growth [6,26]. For instance, in *F. oxysporum*, the MAPK Fmk1 is essential for plant infection and the development of vascular wilt. Mutants lacking *fmk1* display pleiotropic defects, such as altered surface hydrophobicity, impaired invasive growth, and markedly reduced expression of *pl1*, which encodes a critical pectate lyase involved in cell wall degradation [6]. Similarly, orthologous MAPKs—including Mgv1 and Gpmk1 in *F. graminearum* and Fvmk in *F. verticillioides*—have been shown to regulate fungal fertility, development, and virulence [4,36]. Our RNA-seq analysis revealed significant downregulation of a gene encoding an osmoregulatory MAPK in the ΔFW-NPS6 strain. This finding leads us to propose that NPS6 may modulate the MAPK signaling pathway. This downregulation may disrupt the signal transduction necessary for successful host colonization, thereby directly contributing to the attenuated virulence observed in the ΔFW-NPS6 mutant. The production of toxic secondary metabolites is a well-established virulence strategy for many fungal pathogens [33,37]. Our transcriptome data revealed that *FtmPT1*, a dimethylallyl tryptophan synthase that catalyzes the first committed step in ergot alkaloid biosynthesis, was downregulated in the ΔFW-NPS6 strain. This intriguing association raises the possibility of a link between NPS6 and the biosynthesis of secondary metabolites with phytotoxic properties. The precise mechanism by which NPS6 influences *FtmPT1* expression and the potential contribution of ergot alkaloid-like compounds to FON pathogenicity are currently unclear and represent a compelling avenue for further investigation. Taken together, the downregulation of key virulence-associated genes provides a direct explanation for the ΔNPS6 mutant’s attenuated pathogenicity: impaired MAPK signaling likely hinders host colonization, while reduced expression of biosynthetic enzymes like *FtmPT* weakens chemical attack.

Integrating our findings, we propose that NPS6 acts as a central virulence regulator in FON through a hierarchical model: its primary role in maintaining iron and redox homeostasis establishes a cellular foundation that enables the proper functioning of secondary metabolite biosynthesis and stress-responsive signaling pathways (e.g., MAPK), collectively governing pathogenicity. Based on this model, we hypothesize that the iron/redox imbalance in the ΔNPS6 mutant directly or indirectly (e.g., through altered metabolite pools or stress signals) impairs the activation of specific MAPK cascades and represses the expression of secondary metabolite genes like *FtmPT1*. This hypothesis can be tested by comparing MAPK phosphorylation states and *FtmPT1* expression levels in the WT and ΔNPS6 strains under controlled iron availability. This study provides a transcriptomic roadmap that defines the NPS6 regulon in FON, highlighting specific candidates for functional dissection. The most immediate research priorities arising directly from our data include the functional characterization of the differentially expressed nonribosomal peptide synthetases (e.g., *NPS7*, *NPS9*, *NPS10*, and *NPS11*), validation of the role of the phytotoxin synthase *FtmPT1*, and elucidation of the mechanistic link between NPS6 and the MAPK signaling pathway. Beyond mechanistic insights, these NPS6-dependent virulence determinants open new avenues for integrated disease management. The core genes and pathways identified here could serve as targets for designing novel mode-of-action fungicides or for developing DNA markers to expedite the breeding of resistant watermelon varieties. Furthermore, understanding how these virulence factors are deployed may inform the selection or engineering of more effective biocontrol agents. Moving from correlative transcriptomic evidence to causal validation of these targets will be essential to translate our findings into sustainable control strategies against Fusarium wilt.

## 4. Materials and Methods

### 4.1. Sampling of Mutant Strains

The wild-type (WT) strain of *Fusarium oxysporum* f. sp. *niveum* race 1, used in this study, was originally acquired from the Jiangsu Academy of Agricultural Sciences, China. This strain was initially isolated from a diseased watermelon plant exhibiting characteristic symptoms of Fusarium wilt. The ΔFW-NPS6 knockout mutant was generated on this WT genetic background. Specifically, the NPS6 gene was deleted via a homologous recombination strategy, wherein the entire coding region was replaced by a hygromycin resistance cassette to create the ΔFW-NPS6 mutant. The successful and correct construction of the mutant was confirmed by both diagnostic PCR and Southern blot analysis. Hereafter, this mutant is abbreviated as ΔNPS6. Consistent with previous findings in other fungal systems, the ΔFW-NPS6 mutant has been previously demonstrated to exhibit a significant reduction in virulence compared to the wild-type progenitor, underscoring the functional importance of this biosynthetic gene in the pathogenicity of *Fusarium oxysporum* f. sp. *niveum* [5].

### 4.2. RNA Isolation, cDNA Library Preparation and Sequencing

The strain was pre-cultured on PDA plates at 28 °C for 7 days. Conidia were harvested and inoculated into 100 mL of liquid PDB (nutrient-rich condition) or iron-limited Czapek-Dox medium (supplemented with 100 µM BPS) in flasks at a final concentration of 1 × 10^5^ spores/mL. Cultures were incubated at 28 °C with shaking at 180 rpm for 72 h to obtain mycelia in the late-logarithmic growth phase under respective conditions. Mycelia were harvested by rapid vacuum filtration, immediately frozen in liquid nitrogen, and stored at −80 °C until RNA extraction. Three biological replicates were prepared for each condition. Total RNA was extracted from both the wild-type (WT) and the ΔFW-NPS6 mutant strains using the TRIzol reagent method, respectively. (Invitrogen, Carlsbad, CA, USA), following the manufacturer’s instructions. RNA integrity and concentration were assessed. Subsequently, cDNA libraries for RNA sequencing were constructed using the Illumina TruSeq RNA Sample Preparation Kit (Illumina, San Diego, CA, USA), strictly according to the standard protocol provided by the manufacturer.

### 4.3. Read Mapping and Gene Annotation

The obtained clean reads were mapped to the reference genome of *Fusarium oxysporum* using the BLAST-based alignment tool (version 2.2.24) provided by the Fusarium Comparative Database at the Broad Institute (https://www.ncbi.nlm.nih.gov/bioproject/18813, accessed on 25 March 2016). Uniquely mapped reads were assigned to genes using feature Counts, and gene expression levels were calculated and normalized as FPKM (Fragments Per Kilobase per Million mapped reads). Functional annotation was performed using the following databases and tools: Gene Ontology (GO) terms were assigned using Blast2GO 6.0; pathway annotation was conducted using the KEGG Automatic Annotation Server (KAAS) https://www.genome.jp/kegg/kaas/, accessed 15 October 2025; and gene enrichment analysis was performed using DAVID https://davidbioinformatics.nih.gov/, accessed 15 October 2025.

### 4.4. Identification of Differentially Expressed Genes

Differentially expressed genes (DEGs) between the wild-type (WT) and the ΔNPS6 mutant were identified using the DEGseq R package (R version 4.5) [25]. The MARS (MA-plot-based method with Random Sampling) model within DEGseq was applied. Genes with an absolute fold change |FC| > 2 and a *p*-value < 0.01 were considered statistically significant. The fold change was calculated by the Wilson‘s method [18]. Gene enrichment analysis was performed by DAVID and predicting novel pathogenic genes.

### 4.5. Quantitative Reverse Transcriptase (qRT-PCR)

T The qRT-PCR reactions were performed on an ABI PRISM 7900HT Fast Real-Time PCR System (Applied Biosystems, Carlsbad, CA, USA) using SYBR Green chemistry. The thermal cycling conditions were as follows: initial denaturation at 95 °C for 30 s; followed by 40 cycles of denaturation at 95 °C for 5 s and combined annealing/extension at 60 °C for 30 s; finally, a melt curve analysis was performed from 65 °C to 95 °C to verify amplification specificity. The 2^−ΔΔCt^ method was used to calculate relative gene expression levels. Primers used in qRT-PCR are shown in Table 3.

### 4.6. Data Access

The sequencing data have been submitted to the Sequence Read Archive (SRP) database under the accession numbers SRP 066642, SRP 066643.

## 5. Conclusions

We utilized an RNA-seq approach to dissect the transcriptomic landscape governed by NPS6 in *Fusarium oxysporum* f. sp. *niveum*. This strategy successfully identified 30 WT-enriched and 36 mutant-enriched candidate genes that are potentially critical for pathogenicity. These genes span multiple functional categories, including amino acid metabolism, siderophore biosynthesis, MAPK signaling, and secondary metabolism, outlining a complex regulatory network associated with NPS6. Future studies, involving targeted gene knockout and functional complementation assays, will be essential to precisely clarify the mechanistic relationships between NPS6 and these identified genes, and to definitively establish their individual and collective roles in the virulence of this economically important pathogen.

Particularly, genes involved in siderophore biosynthesis and MAPK signaling are prioritized for functional validation. We also note that the mechanistic insights proposed here, derived from transcriptomic correlations, require further experimental confirmation.

## Figures and Tables

**Table 1:** The Kyoto encyclopedia of genes and genomes (KEGG) biochemical mappings for genes expressed in WT and ΔFW-NPS6.

KEGG Categories Represented	Number of Genes	
	WT	ΔFW-NPS6
**Metabolism**	134	139
Amino Acid Metabolism	3	2
Biosynthesis of Polyketides and Nonribosomal Peptides	11	12
Biosynthesis of Secondary Metabolites	40	37
Carbohydrate Metabolism	9	14
Energy Metabolism	21	9
Glycan Biosynthesis and Metabolism	7	23
Lipid Metabolism	12	11
Metabolism of Cofactors and Vitamins	3	7
Metabolism of Other Amino Acids	6	9
Nucleotide Metabolism	10	4
Xenobiotics Biodegradation and Metabolism	13	11
**Cellular Processes**	236	235
Behavior	1	2
Cell Communication	40	45
Cell Growth and Death	11	14
Cell Motility	15	17
Circulatory System	9	10
Development	60	8
Endocrine System	51	53
Immune System	14	60
Nervous System	3	20
Sensory System	11	7
Transport and Catabolism	21	9
**Environmental Information Processing**	107	108
Membrane Transport	4	6
Signal Transduction	51	62
Signaling Molecules and Interaction	52	40
**Genetic Information Processing**	37	43
Folding, Sorting and Degradation	13	13
Replication and Repair	9	4
Transcription	10	9
Translation	5	17

**Table 2:** Genes differentially expressed in ΔFW-NPS6 according to RNA sequencing.

Acc. No. Protein Function	RPKM	Fold Change (ΔFW-NPS6 vs. wt)
**Amino acid transport and metabolism**		
FOXG_000585 choline dehydrogenase	82.4	0.09
FOXG_000341 peptidylprolyl isomerase	172.19	0.13
FOXG_000647 homoserine O-acetyltransferase	61.66	0.15
FOXG_000604 phosphoserine aminotransferase	76.75	0.26
FOXG_000834 maleylacetoacetate isomerase	52.19	0.35
FOXG_000478 homocitrate synthase	108.77	0.43
FOXG_175961 homoisocitrate dehydrogenase	55.26	4.1
FOXG_168639 glutamate carboxypeptidase II	30.08	4.73
FOXG_000016 4-hydroxyphenylpyruvate dioxygenase	172.18	5.55
FOXG_000497 basic amino acid/polyamine antiporter, APA family	232.64	7.35
FOXG_195125 methylenetetrahydrofolate dehydrogenase	47.4	8.36
FOXG_134050 cysteine synthase A	31.19	9.24
FOXG_000472 arginine transporter	80.44	10.71
**Carbohydrate transport and metabolism**		
FOXG_000713 maltose permease	59.21	0.11
FOXG_000094 D-lactate dehydrogenase	272.84	0.19
FOXG_00921 MFS transporter	43.14	0.4
FOXG_134128 Hexokinase	51.01	2.64
FOXG_137571 NAD(P)H-dependent D-xylose reductase (XR)	43.37	2.85
Cell cycle regulation		
FOXG_166060 cell cycle control protein tyrosine phosphatase Mih1	54.81	7.47
**Stress response**		
FOXG_000540 zinc-binding oxidoreductase	228.44	3.65
**Growth and Survival**		
FOXG_000472 osmoregulating MAPK	112.19	0.13
FOXG_001738 TNF receptor-associated factor 6	59.48	4.37
Fatty acid and lipid transport and metabolism		
FOXG_001664 extracellular lipase	64.63	2.17
FOXG_000190 extracellular lipase	107.71	3.81
FOXG_130515 acyl-CoA-ligases CPS1	57.76	7.27
Glycan biosynthesis and metabolism		
FOXG_000478 beta-1,6-N-acetylglucosaminyl transferase	299.58	8.56
FOXG_132156 UDP-N-acetylglucosamine pyrophosphorylase	38.29	10.46
**Information storage and processing**		
FOXG_000540 peptide-N4-(N-acetyl-beta-glucosaminyl)asparagine amidase	87.3	0.1
FOXG_000113 isoleucyl-tRNA synthetase	208.94	0.31
FOXG_132712 alanyl-tRNA synthetase	35.22	5.58
**Inorganic ion transport and metabolism**		
FOXG_000973 sulfate permease, SulP family	37.47	0.23
FOXG_001733 phosphate-repressible phosphate permease	61.64	2.09
FOXG_137308 metalloreductase Fre8	43.79	8.47
**Sulfur metabolism**		
FOXG_128562 choline sulfatase	57.89	3.95
**Nitrogen metabolism**		
FOXG_00886 cyanide hydratase	44.19	0.47
**Metabolism of cofactors**		
FOXG_000058 enoyl-CoA hydratase	290.43	0.13
FOXG_000764 type II pantothenate kinase	55.39	0.17
FOXG_001590 similar to Trans-2-enoyl-CoA reductase	224.92	4.26
FOXG_257691 carnitine O-acetyltransferase	42.21	6.36
**Nucleotide transport and metabolism**		
FOXG_000135 adenine phosphoribosyltransferase	204.82	0.24
Posttranslational modification, protein turnover, folding and assembly		
FOXG_131982 GNAT family acetyltransferase Nat4	39.26	3.97
FOXG_168639 glutamate carboxypeptidase II	30.08	4.73
**Regulation of gene expression**		
FOXG_000190 transcriptional adapter 3	187.1	0.11
FOXG_010129 regulatory protein SWI5	33.11	0.17
FOXG_001075 translation initiation factor eIF-5A	33.72	0.36
FOXG_000497 translation initiation factor eIF-4	107.75	0.46
FOXG_165825 small subunit ribosomal protein S2e	57.39	3.95
FOXG_001655 small subunit ribosomal protein S15e	69.77	4.49
FOXG_000047 transcriptional regulatory protein GAL4	117.1	4.85
FOXG_001610 C6 transcription factor	72.49	5.52
**Secondary metabolites biosynthesis, transport and catabolism**		
FOXG_000637 nonribosomal peptide synthetases 4	66.48	0.11
FOXG_236772 nonribosomal peptide synthetases 7	46.6	0.16
FOXG_000786 nonribosomal peptide synthetases 9	52.56	0.12
FOXG_000578 nonribosomal peptide synthetases 10	82.49	0.42
FOXG_144903 nonribosomal peptide synthetases 11	49.24	0.22
FOXG_000848 dimethylallyl tryptophan synthase FtmPTI	45.86	0.11
FOXG_001684 nonribosomal peptide synthetases 2	62.48	3.32
FOXG 000058 short-chain dehydrogenase	109.37	3.59
**Energy production and conversion**		
FOXG 001941 AMID-like mitochondrial oxidoreductase	41.01	0.19
FOXG_000027 aarF domain-containing kinase	117.12	3.47
**Other**		
FOXG_00944 Carboxymethyl ene butenolidase	39.24	0.34
FOXG_000630 membrane dipeptidase	74.69	0.39
FOXG_168476 Bloom syndrome protein	53.11	8.46
FOXG_000341 5-dehydrogenase	188.09	10.46

**Table 3:** PCR primers used for qRT-PCR validation.

No.	Gene ID	Gene Name	Primers Sequences	
			Sense 5′-3′	Anti-Sense 5′-3′
a	FOXG_000135	adenine phosphoribosyltransferase	GACTTGCGCTCCGTCTCGGCGTTC	GACGATCAGCTGCAGCCTTTGC
b	FOXG_000472	osmoregulating MAPK	GCCCGATATCAACATCTCGTGG	GCATTCAGCTTCTAGCTTGAAATC
c	FOXG_00886	cyanide hydratase	CGTGAGAACTCCATGGCTGTCGAC	GATGACAGCACCGTCAGGACCGA
d	FOXG_001075	translation initiation factor eIF-5A	CCGTTCTCTTCAAAGAACTCGA	GGTGTCACCGTCATCGGTCATG
e	FOXG_000047	transcriptional regulatory protein GAL4	GCAAGCATCTCGGCTTGCAA	GCTCTTGGAAGACCTGCTCG
f	FOXG_001684	nonribosomal peptide synthetases 2	CTCTGAACATCGACGCACC	ATGGAATATGTCTGTCGTG
g	FOXG_001610	C6 transcription factor	GGTATGGATCCACAATACC	GGCGCATGATGGTTGTTTC
h	FOXG_130515	acyl-CoA-ligases CPS1	CGCCTCTCAACCCGGCTTACAAG	TGATAAGACGTACTCATGGTTCG
Reference	GenBank: U37499.1	β-actinI	GCGTGACATCAAGGAGAAGC	TGGGCAACGGAACCTCT

## Data Availability

The original contributions presented in this study are included in the article. Further inquiries can be directed to the corresponding author.
